# A Case of Malarial Keratitis

**Published:** 1882-01

**Authors:** E. J. Gardiner

**Affiliations:** Assistant Surgeon to the Illinois Charitable Eye and Ear Infirmary; Chicago


### Clinical Reports.

### ARTICLE VI.

A CASE OF MALARIAL KERATITIS. By E. J. GARDINER, M.D. Assistant Surgeon to the Illinois Charitable Eye and Ear Infirmary.

In the December number of this journal, my friend, Dr. Hotz, published a paper in which this form of keratitis is so truly described that I consider it superfluous to enter into any general description of the disease in this report. My object in publishing this case is to contribute as much as possible to the knowledge of this form of eye disease, of which thus far so little has been written, and indeed of which, until quite recently, so little has been known. I consider the case a very interesting one, because the patient, having been under my care during the whole course of the trouble, I was enabled to watch the progress of the disease from the very first symptoms.

Mrs. S., age 42, of rather delicate constitution, had been under my care for about a fortnight for a bad case of Blepharitis Ciliaris Ulcerosa. I had succeeded in removing the scabs from her lids, and the ulcers which extended along the whole border of both her upper and lower lids, were very nearly cured. I missed her from my office for a couple of days, and when, on the third day, she returned, she informed me that, on the evening of the last day I had seen her, she was suddenly taken with a chill, which was followed by high fever. Having passed a very restless night, she remained in bed the next day. After I had treated her lids she asked me to examine her eye, because something had got into it on her way to my office. I examined the cornea very carefully, with focal illumination, but *nothing*

*could be seen.* A small foreign body was found in the cul-de-sac of the lower lid. The following day she appeared at my office with her eye bandaged, and stated that in the afternoon of the previous day she had been seized with a most violent pain in her right eye; the pain increased toward night, and had not ceased for a moment. When I opened the lids, I found considerable pericorneal injectoin, very intense photophobia, and very profuse lachrymation. On the upper and outer quadrant of the cornea I detected a little grayish vesicle, from which started a little line of grayish infiltration, which extended outward and a little upward, and ended at the periphery of the cornea. Thinking it to be a case of common phlyctenular keratitis, I prescribed a solution of atropine (gr. ij. to ℥ of water)—and dusted a little calomel into the eye. The patient returned the next day, stating that the medicine had given her no relief. The pain was even more severe. On examination I found that the pericorneal injection had increased, and the photophobia was so intense that moderate light could not be borne. With a good deal of trouble, and not a little persuasion, I at last succeeded in examining the eye with focal illumination. I was well repaid for my pains, for the following interesting condition was revealed: The vesicle and line of infiltration I had seen the previous day had disappeared, leaving in their place a linear abrasion and a little ulcer; from the latter started two little bands of infiltration, one of which travelled directly inward for about a millimeter and terminated in a little vesicle; the other, which also led to a little vesicle, travelled downward and inward, and was also about a millimeter long. The form of the whole thing resembled that of a Y, placed in the horizontal direction. Of course there was then no room for doubt. The case was one of malarial keratitis. I prescribed sulphate of quinine—the atropine to be continued. Two days later I saw Mrs. S. She stated that the pain had subsided after the second dose of quinine. The pericorneal injection had considerably decreased, and the eye bore light very well. With focal illumination I found that two little lines of infiltration had started from the two little vesicles (now changed into ulcers), but they appeared as if the process had been cut short by the treatment, because not only were they much shorter than the other lines, but they did not terminate in vesicles as the former ones had done. The abrasion, which extended from the periphery of the cornea to the first ulcer, had filled up, leaving a line of opaque tissue in its place. The healing process continued steadily from this time on, travelling always from the periphery toward the centre. After two weeks nothing but a diffuse opacity was visible, and after two months this also had disappeared.

---

IODOFORM IN THE TREATMENT OF DISEASES OF THE SKIN.—Mr. Frazer has obtained very favorable results from the use of iodoform in various diseases of the skin. It may be readily employed in the form of an ointment of any required strength, mixed either with lard or vaseline. The strength of the ointment made use of has ranged usually from ten to thirty grains of iodoform to the ounce of cerate, but double this quantity can be applied. It has proved a most useful remedy in healing local eczematous eruptions occurring in strumous children and young people, as well as in cases of impetigo. Mr. Frazer also directs attention to the properties it possesses in curing porrigo decalvans. The best results he has as yet attained have followed the application of vesicating collodion over the affected spot and for a short distance around it. Previous to this it is well to epilate all diseased hairs over the spot, and when the blister is healing the ointment of iodoform should be applied night and morning, or oftener; by this treatment the hair soon reappears in a healthy condition.—*British Medical Journal.*

